# A Lacanian Approach to Medical Demand, With a Focus on Pediatric Genetics: A Plea for Subjectivization

**DOI:** 10.3389/fpsyg.2018.02021

**Published:** 2018-11-01

**Authors:** Rémy Potier, Olivier Putois

**Affiliations:** ^1^Centre de Recherches Psychanalyse, Médecine et Société CRPMS (EA 3522), Université Paris Diderot, Paris, France; ^2^Université de Strasbourg, SuLiSoM EA 3071, Strasbourg, France; ^3^Department of Psychiatry, Mental Health and Addictology, Strasbourg University Hospital, Strasbourg, France

**Keywords:** genetics, pediatrics, demand, subjectivization, medical knowledge, transference, desire, doctor-patient relationship

## Abstract

Current psychological research on contemporary medicine, and in particular genetics, often targets the underpinnings of patients’ attitudes and behaviors with respect to biomedical knowledge and healthcare practices. But few studies approach these underpinnings as manifestations of the unconscious, while so doing could (in particular) help understand patients’ apparent difficulties to understand information, and to subsequently act accordingly (e.g., in making therapeutic decisions, etc.). We hypothesize that Lacan’s (1966) remarks (“The place of psychoanalysis in medicine”) on the transferential nature of the demand addressed by the patient (or his family) to the doctor can help account for these issues: demand filters medical information received from the practitioner, and thereby motivates subsequent decisions. In this paper, we try and shed light on this thesis, and focus on pediatric genetics. We start by describing the manifest doctor-patient-family relationship in the pediatric genetics consultation, in order to show where unconscious determinants can come to play a role (1). We then explain Lacan’s theory of demand: what the patient unknowingly demands is knowledge (*savoir*), the object of which is the body of jouissance – the libidinal experience of one’s body through the first libidinal exchanges with the Other of early infancy, whereby the subject is assigned by the Other (subjectification) a specific fantasmatic status organizing his desire. Patients’ understanding and attitudes thus vary so greatly because of this pre-existing filter. Healing and cure are merely apparent objects of the medical demand, which is an invocative drive seeking knowledge on the cause of one’s desire: medical demand is an instance of transference. Doctors should thus enable patient subjectivization, i.e., help them realize that their demand’s genuine object lies in their pre-existing subjective coordinates (2). In pediatric genetics, apparently paradoxical family attitudes heavily draw on what G. Raimbault, drawing on Lacan, called implicit demand, the object of which is knowledge about the family fantasy giving shape to the guilt of possibly transmitting the disease. We give a clinical example, then show how the concept of demand helped us elaborate the core of a research project on the subjective effects of a genetic deafblindness handicap (3).

## Introduction

One of the main axes of contemporary psychological research on healthcare and biomedicine revolves around the impact of personalized medicine. This is especially true with respect to medical genetics and genomics, which are undergoing an exponential development. This development gives rise to new problems, such as the use of unsolicited or secondary findings, supplemental information unrelated to the patient’s initial request, and yet of potentially crucial medical importance (such as BRCA 1 or 2 – see, e.g., Christenhusz et al., 2013).

In the 2000s, psychotherapists and family therapists were already aware of the need to reflect upon the consequences of this emerging state of affairs:

“When we go for a routine physical, rather than making blanket pronouncements about increasing exercise, lowering cholesterol, and other preventive health measures, our physicians and nurse practitioners are likely to draw individualized blueprints of personal risk factors based on our specific personal genotype” (McDaniel, 2005, p. 27).

The question raised by this state of affairs is: what are the personal, family and social effects of the possibility to receive individualized medical recommendations based on an unprecedented knowledge of one’s genetic and genomic characteristics?

To answer this question, social science research has explored at length the personal and family effects of contemporary medicine (cf. e.g., James et al., 2006; Hens et al., 2016), including the indirect constraints embedded in genetic healthcare pathways (e.g., Vailly, 2013). Within psychology, this question has been scrutinized by cognitive-behavioral psychology (e.g., McDaniel, 2005) or systemic approaches, but few studies have addressed it through the lens of psychoanalysis, with the exception of e.g., Feissel-Lebovici (2001), Aubert-Godard (2005), Driben (2011), Gargiulo et al. (2017). Yet, the originality of psychoanalysis lies in that it can spell out the unconscious determinants at play in the reception of medical information (see e.g. Balint, 1957; Debray, 1996; Gutton and Raimbault, 1975; Raimbault, 1975; Sausse, 1997), of which genetic information is a subset. The specificity of a psychoanalytic approach to this question lies in its grasp of how apparently remote autobiographical elements and repressed childhood situations influence the very thought processes of information understanding, by structuring the individual’s personality up to the very way in which she asks for help and assistance – and what she thereby genuinely expects.

Therefore, psychoanalysis can shed an original light on two pressing issues which, albeit encountered daily in clinical practice, are rarely dealt with directly in research papers, especially outside of French-speaking psychoanalytic literature:

(1) the unconscious determinants of patients’ difficulties to understand genetic information; and
(2) the unconscious determinants of subsequent attitudes or behavior disregarding (or even contradicting) recommendations based on this information – e.g., in taking therapeutic decisions, from short-term life-or-death transplant to long-term therapeutic compliance.

While biomedical and genetic knowledge have developed exponentially since Lacan’s (1966) lecture at the Salpêtrière Hospital (entitled “The Place of Psychoanalysis in Medicine”), we believe that the theory of the demand in the medical field laid out in this lecture can be of help in spelling out the unconscious determinant(s) at play in the reception of genetic information.

Some of the literature partly addresses such unconscious determinants upon the reception of medical information in a Lacanian fashion, e.g., in the French-speaking psychoanalytic tradition (Del Volgo, 1997; Brun, 2005 gathers important collective proceedings on this topic; Lebrun, 2017; Weber, 2017). But we would like to approach them from an angle which, to our knowledge, hasn’t been explored as such – especially in genetics – that of the concept of demand^1^ (We leave aside non-Lacanian approaches of demand in medicine; integrating them would require a systematic review).

Thus, our goal will be to provide a presentation of the Lacanian approach of demand, and to explore how it can be drawn upon to understand the clinical stakes of pediatric genetics. As we shall see, the interest of this specialty is that the unconscious dynamics (aimed at by the notion of demand) implicitly at work in the background of what is explicitly asked of the medical practitioner, come more readily to the forefront: it is generally parents who come for their child’s disease – this leads them to express how they unconsciously represent their child. This family context thus helps shed a strong light on the weight of the unconscious fantasies at work in parental demand, which bear on the psychical appropriation of the information and subsequent decision-making.

In fact, the present paper presents a research trajectory, from the experience of partaking in pediatric genetics consultations within a renowned clinical genetics unit (Imagine Institute, located at Necker Hospital in Paris), to the elaboration of a funded research project on the psychosocial determinants of the impact of genetic deafblindness (DéPsySurdi, see section “Subjectivizing the Demand in Pediatric Genetics: Clinical Practice and Research Perspectives”). The methodological constitution of this project is the result of the present work on demand, which represents its preliminary stage in many respects.

We first provide a description of the manifest doctor-patient relationship in the pediatric genetics consultation, in order to point where unconscious determinants can come to play a role. We then develop Lacan’s understanding of the demand in medicine – that is, in the patient–doctor relationship. We then apply this understanding to pediatric genetics, by focusing on what Raimbault, a pupil of Lacan’s, called “implicit demand”; and we show how it this concept formed the starting point of the aforementioned research project.

Our central idea is that Lacan’s understanding of the demand, the genuine object of which isn’t medical information and/or healing but knowledge of one’s fantasies about what takes place in one’s body, allows for what we propose to call subjectivization – that is, an awareness that the core object of one’s demand lies elsewhere than in healing or care. Subjectivization accounts for the apparent discrepancy between the information explicitly received to the patient and his family, and their understanding and subsequent actions.

It is by taking into account this unconscious search for another knowledge at work in the patient’s demand that the medical practitioner will be in a position to both enable moments of subjectivization, and deliver an adjusted medical response (both in tone and in content) without being unknowingly caught in the patient’s implicit demand.

## The Manifest Doctor-Patient-Family Relationship in the Pediatric Genetics Consultation

This description of the pediatric genetics consultation derives from OP and RP’s participation to routine clinical consultations in the pediatric genetics unit of Necker-Enfants Malades Hospital (Paris), and subsequent exchanges with medical practitioners in the context of these consultations. In other words, material in this section is not derived from research projects or investigations, but from routine practice.

In France, Necker Hospital has always been at the forefront of an interdisciplinary dialog between medicine and psychoanalysis – both in medical genetics and in child psychiatry. At the time when Lacan examined “The Place of Psychoanalysis in Medicine” (1966), one of his early followers, Ginette Raimbault (M.D., Ph.D., psychoanalyst, who introduced Balint groups in France along with her husband Emile Raimbault) was head of an INSERM (French National Institute for Mental Health) unit working on hereditary child metabolic diseases. Since then, the interaction between psychoanalysis and pediatric genetics at Necker has been constant: many consultations are carried, on an ordinary basis, by a pediatrician-geneticist and a psychoanalyst, who contributes to the consultation as he sees fit (and can, if needed, meet with patients afterward).

Classical medical genetics is mostly concerned with Mendelian inheritance of pathogenic variants (along with random spontaneous mutations, called *de novo*); as such, it mostly focuses on monogenic diseases – accounted for by the variation of a single gene – or, at broadest, on a defined set of genes. Pediatric genetics is thus the best setting for psychoanalytic work on the personal impact of genetics: since it revolves around Mendelian transmission, its effects can be best witnessed in clinical contexts where families come to the Medical Genetics Unit to sort out both the name and the cause of their child’s disease.

This is typically a three-step process: first a clinical examination (comprising the proposition to undergo genetic sequencing and, in case of acceptance, the signature of an informed consent form), followed by sequencing (genetic analysis, on the basis of questions raised by the clinical examination), and then – a couple weeks later – by the announcement of the diagnosis (or lack thereof), along with therapeutic advice (if possible).

A specific trait of pediatric genetics is that clinical examination involves questions regarding potential antecedents in family history: the geneticist, in addition to undertaking a clinical examination of the child and questioning his parents, searches for signs of the disease in previous generations and relatives while drawing a family tree. This entails that the explicit parental demand to the practitioner directly puts parents themselves in a position to receive confirmation that they have transmitted the disease – if the genetic character of the disease hasn’t been established already. This context cannot but trigger family guilt: whatever the results of the analysis, the anxiety to have passed on the disease is in everyone’s minds – to the point, not infrequently, of inducing momentary psychical splittings, as when parents, e.g., leave out of the family tree a deceased relative who happened to have signs of the disease.

After sequencing (biological analysis), another consultation is planned for the announcement of the diagnosis. It is often extremely emotional, due to the guilt-laden anticipation – conscious or not – of having in fact transmitted the disease: learning that the child indeed has a genetic disease would be synonymous with having passed it on to him, news which can sometimes trigger deferred psychotic or psychotic-like onsets – be they momentary or revealing a personality structure – if parents are fragile. (In *de novo* cases, where the child is the first to have the disease because of a spontaneous mutation in the parents’ sexual cells, we often witness guilt as well, but in a reversed form, so to speak: parents feel guilty because their child is the only one affected with the disease.)

A striking feature of such consultations is that, after the practitioner has taken the time to announce the diagnosis, and then given information about the transmission of the disease (dominant vs. recessive, etc.) and the therapeutic and lifecourse implications for the child, parents often have great trouble making sense of the medical information they have received – be it immediately or, more frequently, shortly afterward. Often do the geneticists find themselves in a position to have to explain again the mode of transmission and its implications, up to a point where it clearly appears that the real question isn’t “what is the disease and how has it been transmitted?,” but “Why us ?” – in other terms, an attempt to make sense of blind biological fatality. The geneticist is the bearer of bad news, his speech is very often received as an oracle-like prediction (Feissel-Lebovici, 2001; Munnich, 2014); yet, even when he has successfully isolated the pathogenic variant, parents are often perplexed and cannot make sense of these traumatic news. This is often evidenced in their spontaneous question about what can now be done to cure their child – while it has just been made perfectly clear to them that only symptom-oriented care (at best) could hereafter be implemented.

Geneticists experience the same type of perplexity during follow-up consultations about medical decisions and care: often do they see that the previously communicated (and repeated) information concerning the stakes of proper therapeutic decisions doesn’t seem to lead the parents to what would, from the outside, appear as the most reasonable decision – such as transplant, choice of medically adequate treatment, etc.

For example, in pediatric immunogenetics, it is not rare to see parents refusing life-saving bone-marrow transplants for their children, because of the residual 10% risk of lethal outcome – while, by refusing, they could be seen as in fact becoming responsible for their children’s future death, bound to happen if the immune system keeps deteriorating for genetic reasons.

How can a Lacanian approach to the patient–doctor relationship taking place in pediatric genetics account for this often paradoxical gap between the objective, medical information transmitted to parents and patients, and its subjective reception and elaboration? We first need to lay out Lacan’s understanding of the demand in contemporary medical consultations (2). We will then use these elements to explore how they come to play in pediatric genetics (3).

## The Meaning of the Demand in the Contemporary Medical Consultation

### A Demand for Knowledge About Jouissance of the Body

In his remarks on “The Place of Psychoanalysis in Medicine” (Lacan, 1966), Lacan writes that psychoanalysis can help medical practice – *and is, in this perspective, part of it* – since it can spell out what is at stake in the “authentically medical position” (Lacan, 1966, p. 301): namely, the mode of response to what the patient unconsciously expects from the doctor, through what Lacan calls “the demand” (id., p. 302).

Paradoxical as it sounds, the patient’s doesn’t primarily expect healing, which can be provided by therapeutic devices and agents (surgery, drug, etc.). Aside from healing, “a certain something remains constant, and every doctor knows what it is”: demand. And “the significance of the [patient’s] demand, wherein the medical function authentically comes to play” (id., p. 302), is that it is a “demand of knowledge.” That is, the demand to the medical practitioner is an instance of what psychoanalysis calls *transference* (Lacan, 1966, p. 308), whereby the subject supposes a knowledge in the addressee of his demand, thereby considered as a “subject supposed to know” (Lacan, 1973, Chap. 18; various texts in Brun, 2005 refer to this point).

The object of this type of knowledge is *not* the body defined as what can be “photographed, X-rayed, calibrated, diagrammatized” and so on (Lacan, 1966, p. 303), by the medical devices which help establish the diagnosis and heal. In other terms, the body is not to be understood, in the footsteps of the “Cartesian dichotomy between thought and extension” (id.), as a highly complex machine, in spite of the exponential development of exploration and imaging devices which present a purified version of it (cf. e.g., Potier, 2009). One should be aware that this exponential development fostered an “epistemo-somatic rift” (Lacan, 1966, p. 303) encouraging to (mis)understand the body (*soma*, in greek) upon which medical knowledge (*episteme*) should focus – and to miss that it is not to be understood as a complex machine, but as a nexus of “jouissance” (id.). This rift is typical of contemporary medicine: the diversity and complexity of healing devices, machines and substances developed on the basis of biomedical scientific progress tends to overshadow the specific function of the practitioner, whose very authority and personal prestige were deemed throughout the ages to be a central part of his function (Lacan, 1966, p. 297).

What the patient demands from the doctor as subject supposed to know is a knowledge about the jouissance taking place in his body. “The rapport thanks to which the doctor is what he is, is the patient’s demand. Inside this strong relationship where so many things take place, this dimension is fully revealed in its original meaning (…): the relation to the body’s jouissance” (Lacan, 1966, p. 309).

We thus need to briefly account for the constitution of the subject’s relationship to the body’s jouissance, in order to shed light on the patient’s demand to the doctor.

### The Subjectification of Jouissance: Drive, Demand, and Desire

In this context, jouissance refers to the untamed, not-yet-organized circulation of excitation which takes place in the infant’s body during the primordial interactions with his human environment, whereby the infant experiences his body as such (Lacan, 2016, Chap. 13). It is a pure erotic experience of one’s organic being, in all its intensity – a jouissance of being (cf. also Dimitriadis, 2017) [It should be noted that while this jouissance involves direct interactions with the Other as real, since it corresponds to a “mythical” (Lacan, 2016, Chap. 13) moment prior to the linguistic constitution of the subject *qua* separated – more on this just below, the Other is correspondingly *not* experienced as separated, but as part of a field of jouissance comprising himself and the infant].

At this mythical (i.e., reconstructed) stage of the constitution of the subject, in the infant’s state of absolute dependence upon its environment (Freud’s *Hilfslosigkeit*), it is the Other’s *response* to the bodily manifestations of anxiety to which jouissance gives rise which retroactively converts these manifestations into an appeal. This is the first step of the process of subjectification (*subjectivation*, Lacan, 2016, Chap. 12): the infant’s alienation to the Other’s response.

The paradigm case is the infant’s cry (cf. Lacan, 2016, Chap. 24): it is the “marks of [the Other’s] response that had the power to turn his cry into a call” (Lacan, 2006, Remarks on Daniel Lagache’s Presentation). While the infant’s cry doesn’t initially express a specific need (since he wouldn’t know what he needs), but instead manifests an unbearable excitation and is thus at the level of jouissance, the Other (typically, the caregiver) interprets it as a call for a specific action on his side – which will, in turn, be determined by how He unconsciously represents the infant. This representation is constituted by signifiers, discrete elements of speech considered as distinct sounds, independently of their usual socially determined meaning; the specific signifiers which constitute the Other’s representation of the infant will form the latter’s ego-ideal, the very core of his subjectivity.

These marks, in which the all-powerfulness of the response are inscribed, are thus circled in reality with the signifier’s line. It is not without reason that these realities are called “insignias.” The term is nominative here. It is the constellation of these insignias that constitutes the subject’s ego-ideal (Lacan, 2006, Remarks on Daniel Lagache’s Presentation).

That is to say, the function of the Other’s response is to enable a primary identification to bind the infant’s jouissance through signifiers which represent him for the Other. Herein lies the “all-powerfulness of the response.”

The function of the Other’s initial response is thus to turn the bodily jouissance of the cry into what Lacan calls an “invocative drive” addressed to the Other (Lacan, 1973): by understanding the cry as a call, the Other leads the infant to experience what takes place in his body as a drive (with its source in a specific erogenous zone, the mouth), aimed at satisfaction and expressed as a demand. The cry thus becomes “the radical knot where demand and drive come to be bound” (Lacan, 1973, session of May 27^th^, 1964 – modified translation).

At this level of primordial alienation, where the infant *qua* subject of jouissance is bound to grasp what happens in his body through the response of the Other, he undergoes an identification to what Lacan calls object a (*objet petit a*) of the Other, wherein he comes to wonder “what the Other wants from him” (Lacan, 2016) in so responding to his cry.

Therefore, he needs the Other to elucidate the signifiers of primary identification (often written S_1_ by Lacan) by drawing on a constellation of complementary signifiers (written S_2_) that account for the Other’s choice of S_1_. Typically, S_2_ stands for the Oedipal narrative which accounts for the unconscious choice of S_1_ by the Other – most often the mother. [In most cases, the maternal or mothering Other will be in a position to provide such a constellation by drawing on the Name-of-the-Father, Lacan’s formal re-writing of the Oedipal complex (Lacan, 1998); for a more detailed recent presentation, cf. e.g., Razon et al., 2017, see section “The Manifest Doctor-Patient-Family Relationship in the Pediatric Genetics Consultation.”) In such a second step, whereby the primordial Other is divided by the necessity to account for his choice (most often by leaving room in the S_2_ for another figure co-defining the infant’s identity through a paternal function, such as the father), the *object a* to which the infant was identified acquires a new meaning through S_2_ – and the infant can thus know what he is for this Other, i.e., what the Other wants from him (Lacan, 1973, 2016). From the perspective of the Other, the infant becomes an object of *desire* since he is viewed as a representative of *another desired figure*; he becomes, as Lacan puts it, “phallicized” (Lacan, 1973). From the perspective of the infant, the maternal Other thus appears as desiring, since she also cathects someone else, who partly accounts for what the infant represents for her. Lacan calls this second step “separation” (Lacan, 1973).

Thus, at the end of this reconstructed two-step process of unconscious subjectification by alienation/separation, the cry has become a demand *qua* invocative drive. Correspondingly, its object, i.e., what could genuinely satisfy it, isn’t merely the oral partial object (breasts, etc.). Since the maternal Other, when giving the breast to a crying infant, draws on the signifier-based framework of Her representation of the infant qua object a of desire, *it is Her repressed representation of the infant qua object a, which constitutes him as subject of the unconscious, which is the object of his demand.*

Thus, once the subject is constituted, everything that he comes to voice will, from the perspective of the unconscious, have to be understood as a demand, unknowingly articulating the signifiers which constitute the coordinates of the particular *object a* that he is for the Other.

### Consequences on the Patient–Doctor Relationship: Subjectivizing the Demand

It is for this reason that Lacan starts his conference on “The Place of Psychoanalysis in Medicine” by stressing the “gap between demand and desire” (Lacan, 1966, p. 302): while the manifest demand addressed to the practitioner looks like a demand for healing, the repressed signifiers of the desire of the Other to which the demand can be related show the discrepancy between what he demands and what he genuinely desires.

When he is sent to the doctor, or comes to meet him, the patient does not simply expects to be healed. He puts the doctor to test, to see whether he can bring him out of his condition; this is altogether different from healing the patient, since this demand can imply that the latter very much wants to remain ill. Sometimes the patient wants us to authenticate his status of illness; in many other cases, his obvious wish is that we help him remain ill, treat him in the way he wants, which will help him remain settled within his illness. I just need recall a recent experience: a patient, who recently came in a formidable state of permanent anxious depression having lasted for more than 20 years, was in utter terror at the idea that I could do something for him.

(…) As soon as we’ve pointed out [the gap between demand and desire], it appears that it isn’t necessary to be a psychoanalyst, nor even a doctor, to know that once anyone, be they our best friend, male or female, demands something, it is in no way identical to – and, sometimes, in full opposition with – what they desire (Lacan, 1966, p. 302).

What the patient desires can thus, depending on the structure of the signifiers which constitute him as subject of the unconscious, amount to various types of relationship with the Other – such as, e.g., remain dependent from Him (“help him remain ill”) – which are then projected onto the person of the medical practitioner. These types of relationship with the Other refer to the type of object *a* to which the subject is reduced by the desire of the Other – this is the formula of the fundamental fantasy (Lacan, 1973), which formalizes the role and the organ (mouth, etc.) to which the subject identified at the step of separation from the Other. It is this formula, to which the subject identified in separation, which gives its shape to the desire of the Other, and that the subject unknowingly seeks to uncover by voicing his demand, which is at bottom transference, i.e., a “demand of knowledge” (Lacan, 1966, p. 308).

Hence the importance of the medical response: strictly understanding what the patient says as a demand for healing via a cure, and thereby missing that the signifiers used or hinted at by the patient are indirectly referring to something else (the object *a*) will prevent the doctor from grasping that what he wishes is to know the truth about the structure given to his jouissance by the desire of the Other, i.e., about the fantasy at play.

Correspondingly, it is by taking into account this unconscious search for another knowledge at work in the patient’s demand that the medical practitioner will be in a position to deliver an adjusted medical response (both in tone and in content). In the medical consultation, especially in the context of heavy medical examinations, leaving out this dimension will typically lead the patient to persist in fulfilling his unconscious role in the fantasy (e.g., request more and more examinations, or act in opposition with what he is told). Reversely, the medical consultation (as Del Volgo, 1997 has insisted) provides the practitioner with a context propitious to help the patient gain awareness, and question the consistency, of the knowledge of his jouissance that he supposes that the Other holds – in a movement analogous to the end of a psychoanalytic cure, where transference is dissolved, i.e., the consistency of the subject supposed to know collapses (Lacan, 1968(unpublished), Session of January 10^th^, 1968). “On the one hand, [the doctor] deals with an energetic cathexis, the potency of which he cannot suspect if he isn’t told about it” – i.e., transference – and “on the other, he needs to put this cathexis between brackets precisely because of the power that he possesses, that he needs to distribute [i.e., medicine, OP], and of the scientific plane within which he is situated” (Lacan, 1966, p. 308). In so doing, he puts his medical knowledge between brackets in order to gain access to the patient’s representation of his knowledge about jouissance, *in order to be able to provide the right, adjusted medical response*.

This analysis of the medical function thus implies that it depends on the doctor to hear the patient’s demand as the manifestation of a desire to know something about his jouissance: he can thereby help the patient become aware of his desire, instead of responding to the demand solely by drawing on the position granted to him by his knowledge and position. This is certainly not to say that the medical practitioner has to explicitly interpret the patient’s discourse: a medical consultation isn’t a preliminary interview prior to the initiation of a psychoanalytic cure. But being aware that the demand’s object is knowledge upon the patient’s jouissance can help make the latter aware that the truth of his demand (in the psychoanalytic sense of the term: the *subjective* truth) doesn’t primarily lie in medical knowledge – once again, a preliminary step to an adjusted medical response in terms of cure and healing.

We propose to call the awareness that the medical practitioner can help the patient experience a *subjectivization* of the latter’s demand. *Subjectification*, the word aptly chosen by A. Price to translate the French word “subjectivation” (Lacan, 2016, Chap. 12), refers to the *constitution* of the subject through alienation to, and separation from, the Other; we view *subjectivization* as referring to something different, namely the process of becoming aware of the essentially subjective nature of the demand to the practitioner concerning what happens in his body. Subjectivizing means understanding, to some extent, that the meaning of this demand derives from elements of one’s own subjective coordinates; in Lacanian terms, this amounts to understanding that the signifiers of one’s demand have to be referred to the primary signifiers in the Other, which assign the subject to a certain position qua object *a* of the desire of the Other. A medical consultation carried by a practitioner aware of both medical stakes and the subjective meaning of demand, can help the patient partially grasp this subjective meaning, and question what it is he wants from the practitioner.

Focused on producing in the subject an interrogation on the genuine meaning of his demand (and open up the way for a potential further inquiry on this desire itself), subjectivization in a medical context is a the condition for adjusted medical action, and a potential preliminary step with respect to a potential deeper elucidation – such as the one carried in a psychoanalytic cure, which ultimately aims at helping the subject move beyond his assignation as object *a* of the Other’s desire.

### An Instance of a Setting Enabling Subjectivization: The Instant to Say

We can illustrate this concept by commenting an example through which Del Volgo presents the original clinical setting that she calls the “instant to say” (1997, p. 61), which we view as a typical setting enabling subjectivization. Del Volgo, both a hospital medical practitioner and a Lacanian psychoanalyst, gives examples of how, within the context of a medical consultation, she asks patients about their medical history in such a way as to enable an “instant to say.” This refers to a logical moment when patients, by recalling the important events of their life in the course of recounting the history of their illness and its various stages or occurrences, are presented with the opportunity to grasp the signifiers with which they describe the illness *in relation to important prior life events*. While this opportunity isn’t presented explicitly, or as a goal of the consultation, this associative process opens up a space aside the healing-oriented dimension of the medical response, and gives them a chance to grasp and question the meaning of their medical demand – that is, the structure given to their jouissance by prior important life events. In so doing, she doesn’t respond do the immediate demand but tries to help the patient gradually become aware of the subjective significance of his symptoms, i.e., of the fantasy which underlies them.

We will comment on a case that she presents in Del Volgo (1997). An elderly asthmatic patient, Ange, experienced a severe asthma crisis upon learning from the specialist that his wife, after 3 weeks of nocturnal hallucinations which made him feel “lost” much like an orphan, was in fact *not* suffering from a brain tumor, as initially suspected. Being asked to recall important elements in his life, he indicates that he has been repeatedly and unexpectedly been put in the position to be the closest to his mother: his father died in the beginning of World War II, when his older brothers had already left the house. He experienced this as becoming the man in the house – an important signifier for his personal history. His first respiratory crisis occurred at age 30, “the age of adulthood” (where he could go see a doctor, unlike childhood where he was once beat up for doing so): he accidentally started spitting blood during physical effort, which (he says) includes physical intimacy. It thus seems that respiratory problems became associated with fantasies of castration as a punishment for *Oedipal desire*, summoned (in accordance with Freud’s bi-phasic trauma theory) in the context of adulthood and conjugal life. It is as if the guilt of desire (being put in the position of a phallicized object *a* vis-à-vis the Other in the fantasy) could find a somatic expression – castration symbolized as bleeding out during effort; and that, conversely, the presence of the Other was experienced as the approach of a forbidden oral object a, thus causing in his body a symbolic equivalent of castration through hysterical conversion.

This interpretation was confirmed through transference during the next consultation, when he mentioned that a cardiac accident occurred while he was eating sweets on the anniversary day of their first consultation: the reminiscence of the first consultation during the second one, and the structure of the Oedipal fantasy within which he is caught up, accounts for the symbolic equivalence between the forbidden pleasure of eating sweets and becoming intimately close with the mother of childhood. The cardiac accident is thus a transferential replica of his first respiratory problems at age 30, confirming that these series of bodily events can be understood against the background of the way in which his jouissance is structured – namely, through an oral Oedipal fantasy. Those elements constitute the background on which the patient’s associations, supported by Del Volgo’s psychoanalytic listening, shed a partial light during this sequence; it was then up to the patient to subjectivize the connection between these past events and the actual occurrences of respiratory problems.

We now draw on this conceptualization of the demand as carrying a repressed desire open to subjectivization (and open to further elucidation), and we turn to pediatric genetics.

## Subjectivizing the Demand in Pediatric Genetics: Clinical Practice and Research Perspectives

### Explicit vs. Implicit Demand in Clinical Practice

As mentioned above, pediatric genetics is particularly interesting to study the demand at work in contemporary medical practice since the structure of the fantasy which organizes the patient’s desire (and thereby filters the reception of information) is often more readily accessible during the consultation. The reason for this is that the demand for diagnosis and cure is voiced for the child by the parents – the unconscious of whom largely contributed to structuring the child’s – who feel responsible for his disease since it is viewed as hereditary (at least potentially: the cause is sought for in previous generations).

As we mentioned above, the main proponent of applying Lacan’s theory of the medical demand to pediatric genetics was Ginette Raimbault, in charge of research on the unconscious stakes of medical consultation at INSERM (French National Institute for Mental Health), and whose clinical field was a pediatrics unit working on hereditary child metabolic diseases – the precise hereditary cause of which was largely unknown at the time, for lack of adequate sequencing apparatus and knowledge. In 1966, right after Lacan gave his lecture on “The place of Psychoanalysis in Medicine,” she gave a didactic presentation of her research – which consisted in assisting silently to consultations and elaborating on the unconscious dynamics at stake in the family’s demand. This is how she describes these dynamics:

“As early as during the first interview with the medical practitioner, the parents formulate the results of their own research about the etiology of the disease, considered as a trouble. (…) The parents’ formulation shifts from ‘this makes no sense’ to ‘this is the sense we give to this disease”’ (Raimbault in Lacan, 1966, p. 313).

While the subjective sense given by the family to the disease partly depends on the medical antecedents, the lack of information or the powerlessness of medical science (op. cit.), it largely derives from “the elaboration of fantasies concerning the agent of the disease” (Raimbault in Lacan, 1966, p. 313).

“the child’s disease thus seems to reveal the family’s problem and its singular drama, which is actualized in the disease and feeds off of it, but isn’t properly speaking caused by it. The difficulties faced by doctors partly stem from the fact that they only hear the explicit demand (‘Cure this crisis!’) and not the implicit one (‘This is our drama’)” (Raimbault in Lacan, 1966, p. 313).

Any medical discourse concerning this hereditary agent will thus be filtered by a *pre-existing* family fantasy organizing what she calls implicit demand to the practitioner.

The specificity of the notion of implicit demand is that it refers to the parents’ quest for help with respect to a guilt which, albeit coming to the forefront at the *occasion* of the child’s disease, *predates* it. Raimbault’s main clinical finding is thus that the disease is filtered by the “window of the fantasy”: to put it in the Lacanian framework which underlies her work, the disease is experienced by the parents (especially the mother) as a *punishment* for their normal anticipated fantasmatic elaboration of the status of the child qua object *a, prior to any medical condition*. Since this anticipated elaboration – way before the birth of the child – cannot but include an element of repressed guilt (even in neurotic contexts: a child is always partly viewed by both parents as an Oedipal child), the subsequent disease is experienced, through an unconscious displacement, as punishment for the accomplishment of the Oedipal wish to have a child with one’s parent.

The notion of implicit demand thus directly echoes Lacan’s characterization of transference on the doctor as a demand for knowledge upon one’s jouissance, and narrows it down to the context of hereditary diseases: what is implicitly demanded is knowledge about the family fantasy giving shape to their guilt. It is in this wake that Raimbault insists that what matters most, on the side of medical practitioners, is to prevent stereotyped attitudes and responses based on unquestioned personal assumptions concerning what stands as appropriate behavior in those medical situations: they are laden with the practitioners’ personal subjective organization, and would prevent him from grasping the family’s implicit demand (Raimbault in Lacan, 1966, p. 314).

The core of the knowledge that he is unconsciously asked by the family – the object *a* of the family’s demand, so to speak (and it most often is the mother’s, in these circumstances) – concerns the particular structure of the desire of having a child, of which they (unavoidably) feel guilty. Responding to their implicit demand would amount to help them subjectivize this family fantasy.

### An Example of Implicit Demand: A Consultation in Pediatric Genetics

The medical context in which hereditary child diseases nowadays take place is pediatric genetics, wherein such implicit demand unfolds. The following example illustrates elements present within a host of consultations, and comes from OP’s practice of pediatrician-genetician/psychoanalyst dual consultations in pediatric genetics. Only de-identified data were used; therefore, an ethics approval was not required for the use of this material as per the Institution’s guidelines and national regulations. It shows how the explicit demand carries an implicit one, which filters both the reception of information and the subsequent decision-making of patients.

In this context, that of the pediatrician-genetician and psychoanalyst, the difference with both Del Volgo’s setting and Raimbault’s research is that the psychoanalytic perspective is embodied by a specific person (not the doctor), who also actively partakes in the consultation, sometimes to an important extent – when the weight of the implicit demand comes to the forefront. It is not only listening, but also active interventions, which open up a space of subjectivization, i.e., of relating the signifiers of the demand (the explicit demand, in Raimbault’s quote) to those of the underlying fantasy of the implicit demand of the family singular drama. This is sometimes needed in order to shed light on the extent to which this demand filters medical information and subsequent behavior.

The following example is reduced to a few elements for anonymity reasons. A young mother of two adolescents was extremely reluctant to try a bone marrow transplant which could save them both of a rapidly developing disease enabled by a hereditary recessive immunodeficiency. Hearing the unmentioned guilt present in her speech, OP told her “in any case Madam, you are not responsible for your sons’ disease” – in order to stress that she couldn’t know, medically speaking, that mothering them would lead to transmitting them the disease, but that her apparent sorrow might be rooted somewhere else. She replied (thereby illustrating the equivocation enabled by the signifier “guilt”): “what do you mean? I am by no means irresponsible! I’m doing my best here!” Her mastery of French language was more than sufficient to rule out a cognitive explanation for her apparent mistake. In so responding, she showed us how guilty she does feel for their disease, experienced transitively as a punishment for what (in the rest of the consultation, in relation to biographical elements) most likely appeared to be the structurally normal (see section “The Subjectification of Jouissance: Drive, Demand, and Desire”), predating Oedipal fantasy of receiving a child from her father – the paradigm forbidden desire of which, at a certain level, she unconsciously expected the consultation to relieve her, by helping her formulate it. The singular drama was thus that she unconsciously experienced this forbidden desire, upon which becoming a mother largely relies, as directly punished by the disease. It is this unconscious connection, qualifying her relation to her children *qua* phallic objects *a* (because of the Oedipal structure of the Other organizing her unconscious), that she needed to subjectivize; for ultimately, the way to partly soothe this guilt is to start by acknowledging it, which is the object of her implicit demand to medical knowledge about what takes place within her children’s bodies, and therefore filters how she heard OP’s intervention.

Unfortunately, this subjectivization (realizing the relation between her experience of the disease and a guilt of a different origin) was made extremely difficult by the pressing therapeutic context, where a decision had to be made in the near future concerning the bone marrow transplant. In other words, aside the response which she unconsciously sought concerning the fantasmatic cause of what was taking place within their children’s bodies, a healing-oriented response also had to be given her concerning the stakes and urgency of the transplant. Upon hearing about the necessity to soon make a decision concerning this matter, she said she was extremely reluctant to accept it, because of the residual 10% chance of lethal outcome (in spite of the certainty of such an outcome in the absence of transplant). One can wonder whether a masochistic need to be punished for the Oedipal character of her fantasy, which enabled her to represent her children as phallicized objects *a* in the first place, could account for her decision: wouldn’t a lethal amount (inexorable in the absence of transplant) symbolically amount to a paradigm punishment for her forbidden fantasy? In this case, what appeared to be the structure of her fantasy could account for her fantasy, with its masochistic components. Her behavior, seemingly paradoxical with respect to the perspective of healing and cure, thus appears in a new light (see section “Consequences on the Patient–Doctor Relationship: Subjectivizing the Demand”).

### Demand-Based Starting Point of a Qualitative Research: The Subjective Effects of Genetic Deafblindness

Finally, we would like to give a brief illustration of the demand-based rationale of an ongoing qualitative research based on this conception of the demand in pediatric genetics. This multidisciplinary research focuses on the subjective effects of genetic deafblindness on autonomy in child, adolescent and adult patients with Usher, Wolfram and Stickler syndromes (research codename: DéPsySurdi). It is funded by the French Rare Disease Foundation (“Fondation Maladies Rares”); RP is its principal investigator, and it has been made possible by a close partnership with the Reference Center for Genetic Deafness (INSERM – U587, dir. Dr. S. Marlin).

What we briefly present here is the nucleus of the psychoanalytic rationale of this research, jointly conceived by RP and OP on the basis of a Lacanian approach to the demand in the context of genetics (and in particular pediatric genetics). This nucleus is both the result of a research on medical demand with a focus on pediatric genetics, and the basis of the specifically psychoanalytical contribution to the DéPsySurdi project; on this basis, collaborators in psychology, medicine and social science joined in order to turn this nucleus into an exploration of the effects of genetic deafblindness at a psychosocial level. (Our methodology, which we cannot fully unfold here, relies on semi-structured interviews using sign language or tactile sign language, in order to leave as much room as possible for association, and more generally punctual emergences of formations of the unconscious.)

The gene-based Usher, Wolfram and Stickler syndromes gradually affect both hearing and sight up to partial or total auditory and visual deficits, resulting in deafblindness (a specific handicap, wherein large parts of audio-visual compensation is impossible). The effects of this handicap on one’s autonomy appear to vary greatly; it has important psychiatric comorbidities, such as depression due to increased social isolation. We decided to examine the effects of deafblindness on autonomy in child, adolescent and adult subjects because autonomy is centrally impacted by this handicap, and is thus the natural manifest object of family and patient demand: the demand for medical and social help and support greatly focuses on compensating this handicap, especially in parents with children and teens affected with these syndromes. Therefore, various strategies, devices (e.g., technological) and personalized supports (personal or family assistants, etc.) are devoted to this compensation, and thereby help young patients and their parents achieve social participation and self-realization: the explicit objects of the families’ demands are means to ease the burden of the handicap and facilitate interaction.

This initial context raises specifically psychoanalytic questions: doesn’t the variety of available supports sometimes cloud the subjective significance of the syndromes? In other words, behind the need for help and support, does the current available medico-social leave room for subjectivization in the families’ and patients discourse on the handicap? To what extent can they question the signifiers which, at the manifest level, they use to refer to the everyday impact of the handicap, and ways to alleviate its burden? This would mean having the opportunity to relate the gene-based loss of autonomy (along with the parental guilt which accompanies it and is reinforced by genetic sequencing) to what Raimbault called the predating family fantasy which gives its particular sense to their child’s handicap. In this respect, a particularly sensitive question is the degree to which the child’s participation to the family fantasy – and conversely, his degree of autonomy *with respect to it.* What room do they leave for the gap between their child’s autonomy in the expression of his demand, and their own representation of him (laden with the guilt of his syndrome, which clouds the structural predating guilt)? And in cases where this gap is thin, is his demand devoid of singular desire, i.e., just a reflection of his main caregiver’s fantasy, in which he would be caught up? Or does it present aspects of a singular desire of separation *qua* phallicized object *a*, waiting to be acknowledged as such? This stake is particularly crucial, since the context of deafblindness leaves less room for separation, since communicating often requires to use tactile sign language – and thus to touch. What of the potential equivocity of the signifier in these contexts?

These are the questions which led us to decide to focus on this cluster of syndroms, in order to help doctors position themselves with respect to the unconscious question concerning one’s body at play in the demands for support that they receive.

## Conclusion

In this paper, we wanted to draw on Lacan’s take on the demand to stress that the exponential development of personalized and stratified medicine, which help provide previously unexpected adjusted cure and healing, requires medical practitioners to remain sensitive to the dimension of the demand for knowledge about jouissance, in order to prevent their response (cure, investigations, etc.) from reinforcing the underlying repressed fantasies, with their masochistic basis.

It is even more important when the object of this knowledge is what takes place in someone else’s body: these situations are often quite projective, in the sense that it is difficult for caregivers to acknowledge that their understanding of their child’s bodily symptoms is heavily influenced by his role in their fantasy. This is particularly true when the child represents the object of the Mother’s (and not the couple’s) fantasy, as Lacan wrote in the first “Note on the child” in 1969 (Lacan, 1986).

The unconscious guilt of having transmitted the disease, even in cases where transmission cannot be established (which are numerous, even as of today), contributes to these projections. It is for this reason what we chose to focus on pediatric genetics, since this clinical field is both saturated with such projections, but at the same time open to potentially disalienating interventions – especially if children themselves, as well as other members of the family, actively partake in the medical exchanges, so as to distinguish their own speech to their parent’s implicit demand.

## Author Contributions

RP has contributed to part of the material (including clinical) presented in this paper through an uninterrupted dialog with OP during the latter’s post-doctoral research (which he *de facto* partly supervised); he has co-conceived the psychoanalytic basis of the deafblindness research, and is the Principal Investigator of the resulting psychosocial project (DéPsySurdi – http://www.depsysurdi.fr/). He has given useful comments on a previous draft of this paper, and has agreed to the final version. OP has conceived the structure of the paper and its argumentation (topic and organization were based on his post-doctoral research), taken part in most of the consultations mentioned in the paper, has co-conceived the psychoanalytic basis of the deafblindness research, and written the paper.

## Conflict of Interest Statement

The authors declare that the research was conducted in the absence of any commercial or financial relationships that could be construed as a potential conflict of interest.

## Footnotes

1 While we stuck to the usual English translation, the meaning of the French “demande” differs from that of the English “demand,” as will appear below in more detail. While the English “demand” implies a positive requirement, and frequently a dimension of command, the French “demande” (especially in its psychoanalytic understanding) mostly refers to the expression of helplessness – so much so that it often means to beg or to implore.
